# Supplementary figures and images for: Agomelatine prevented depression in the chronic restraint stress model through enhanced catalase activity and halted oxidative stress
Source: PLoS One. 2024 Feb 9;19(2):e0289248. doi: 10.1371/journal.pone.0289248 (PMC10857580; doi:10.1371/journal.pone.0289248)

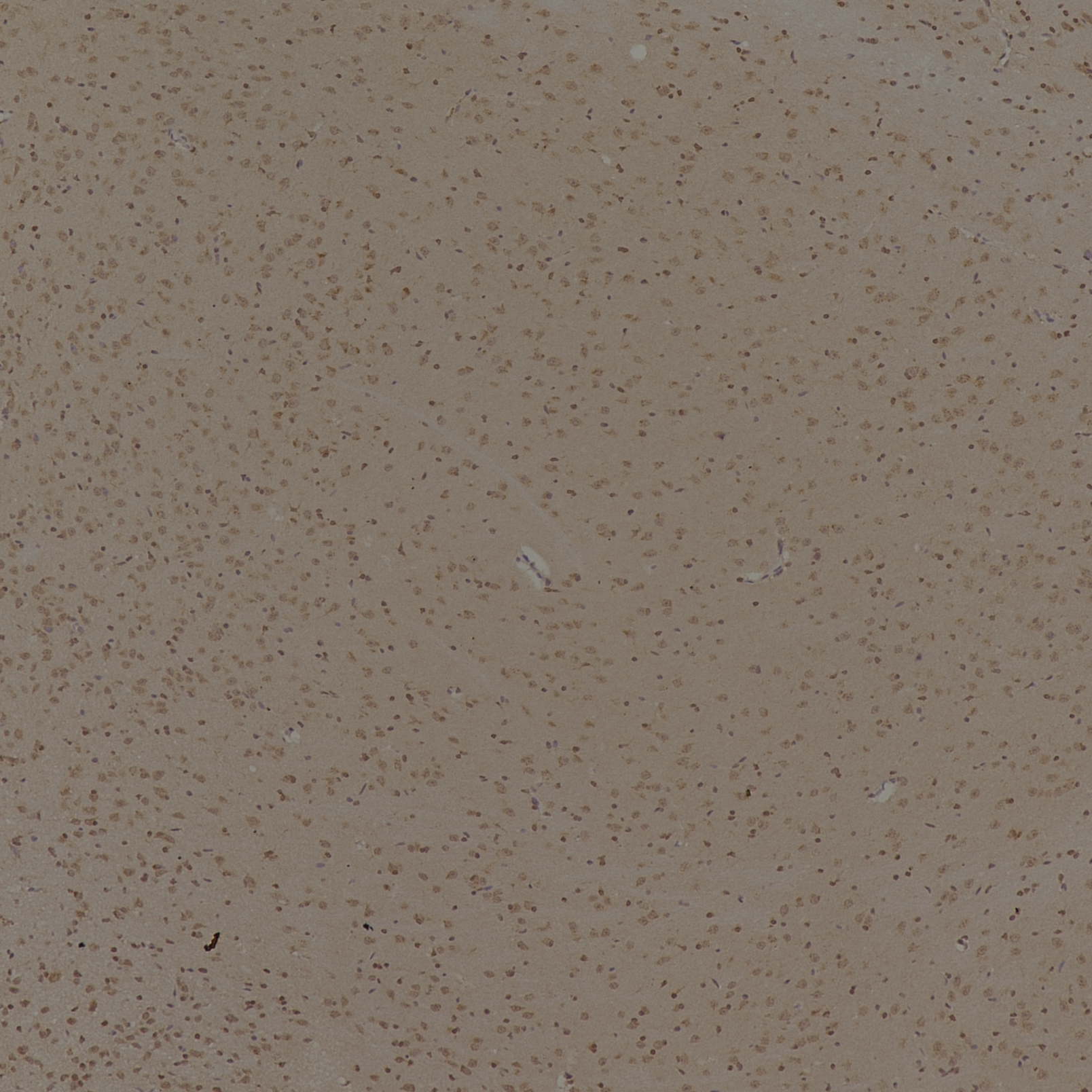

Supplement: S1 File — (ZIP) [file pone.0289248.s001.zip › data in brief/Immunohistochemistry/10x CON for fig.2(a).png]

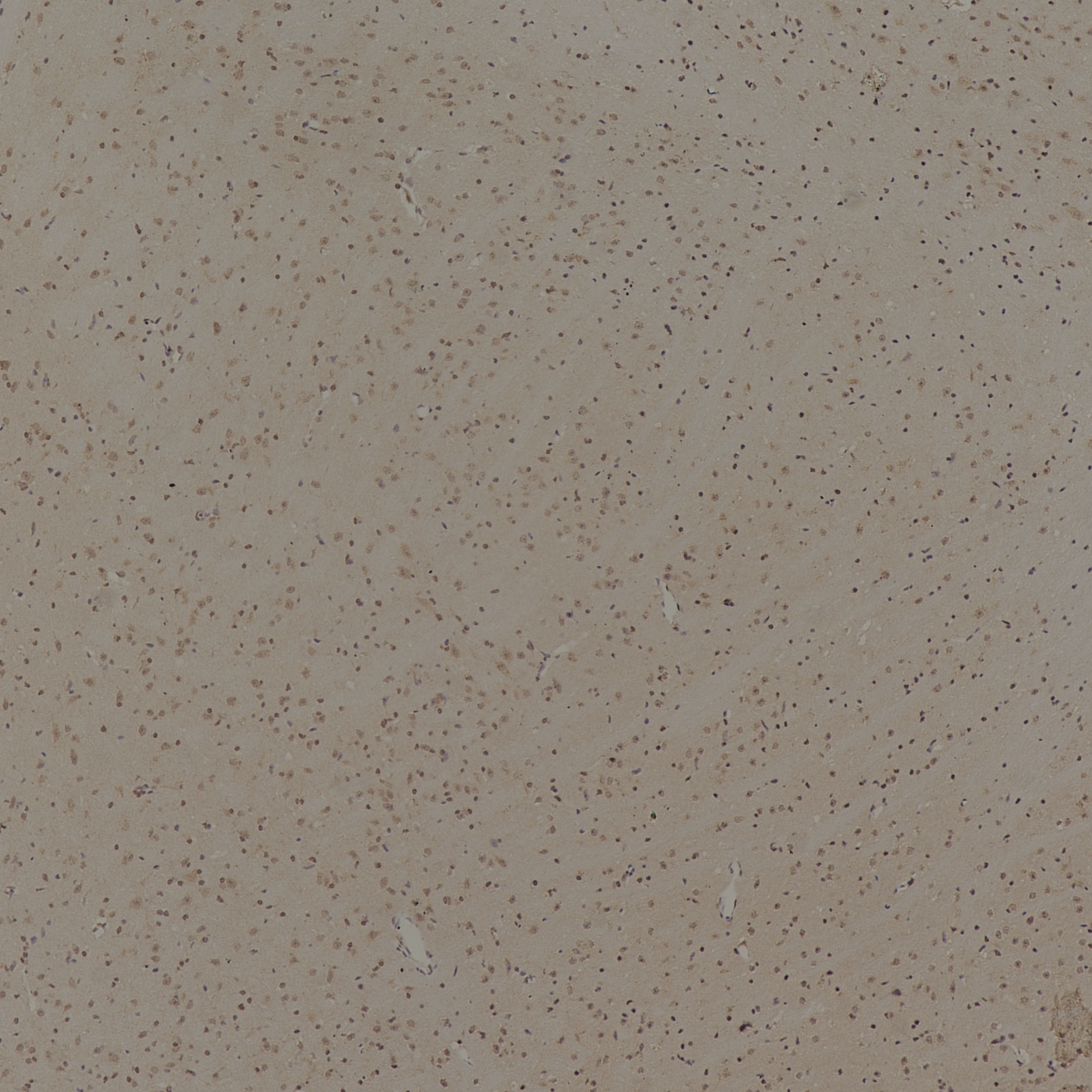

Supplement: S1 File — (ZIP) [file pone.0289248.s001.zip › data in brief/Immunohistochemistry/10x CRS for fig.2(a).png]

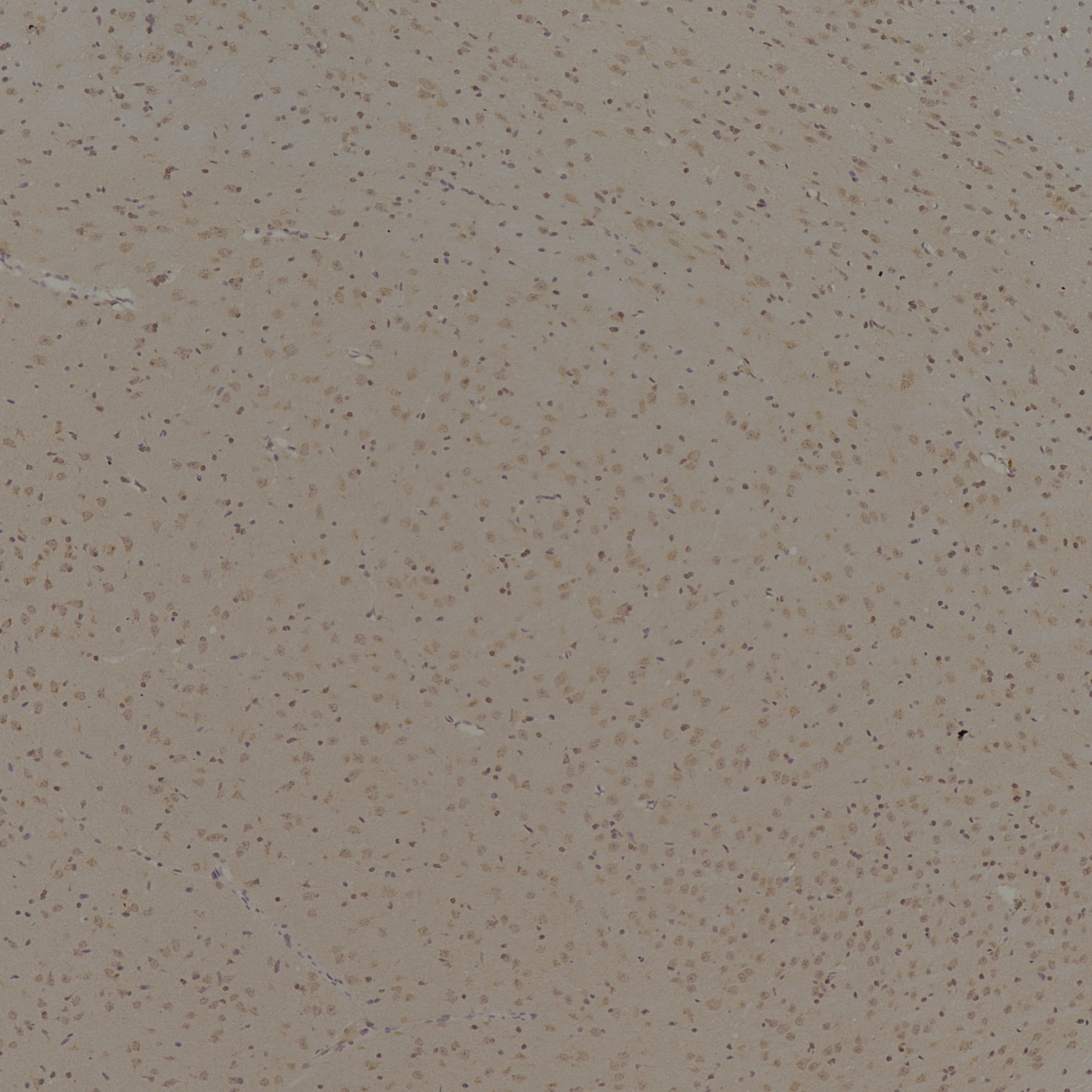

Supplement: S1 File — (ZIP) [file pone.0289248.s001.zip › data in brief/Immunohistochemistry/10x CRS+AGO for fig.2(a).png]

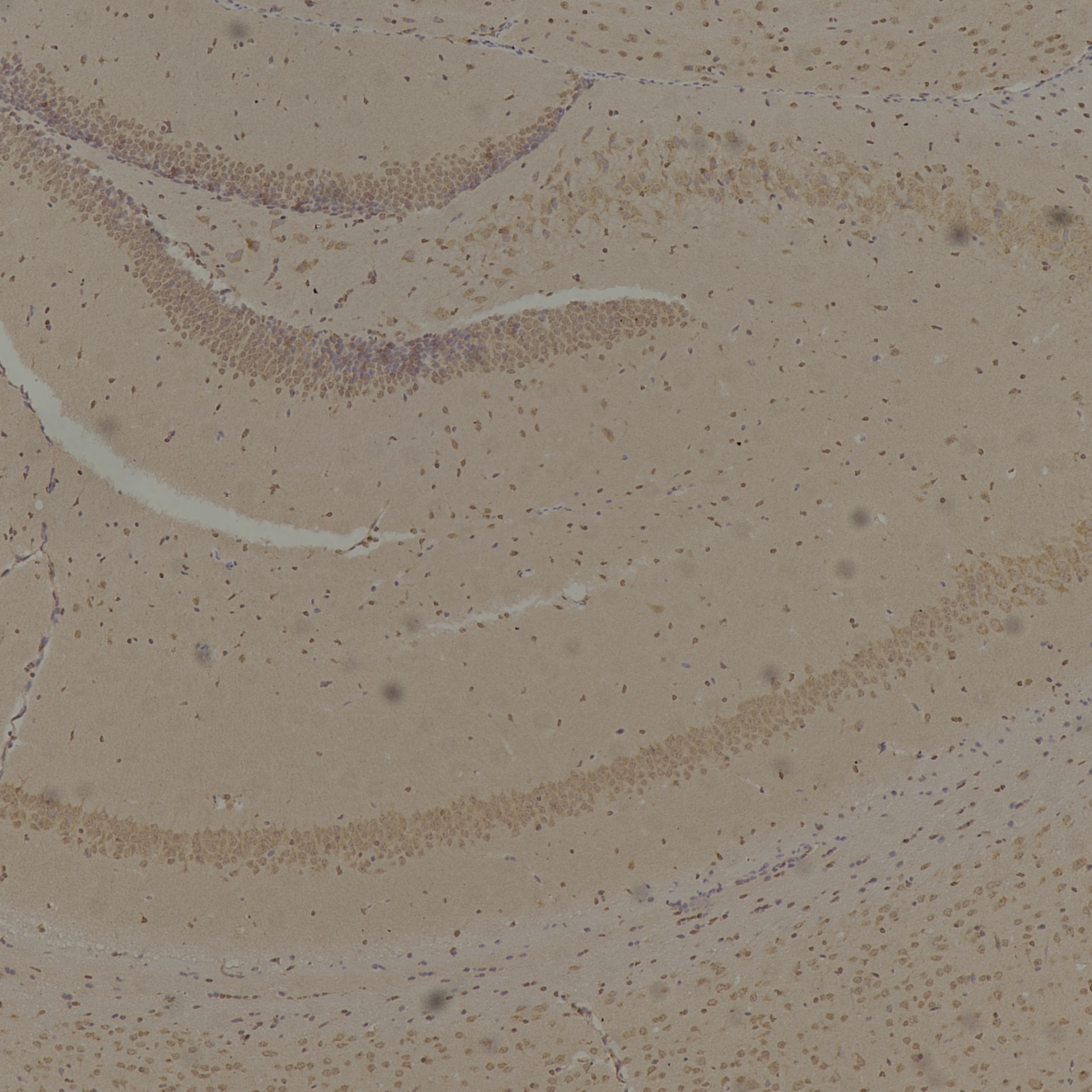

Supplement: S1 File — (ZIP) [file pone.0289248.s001.zip › data in brief/Immunohistochemistry/10x CRS+AGO+DFX for fig.2(a).png]

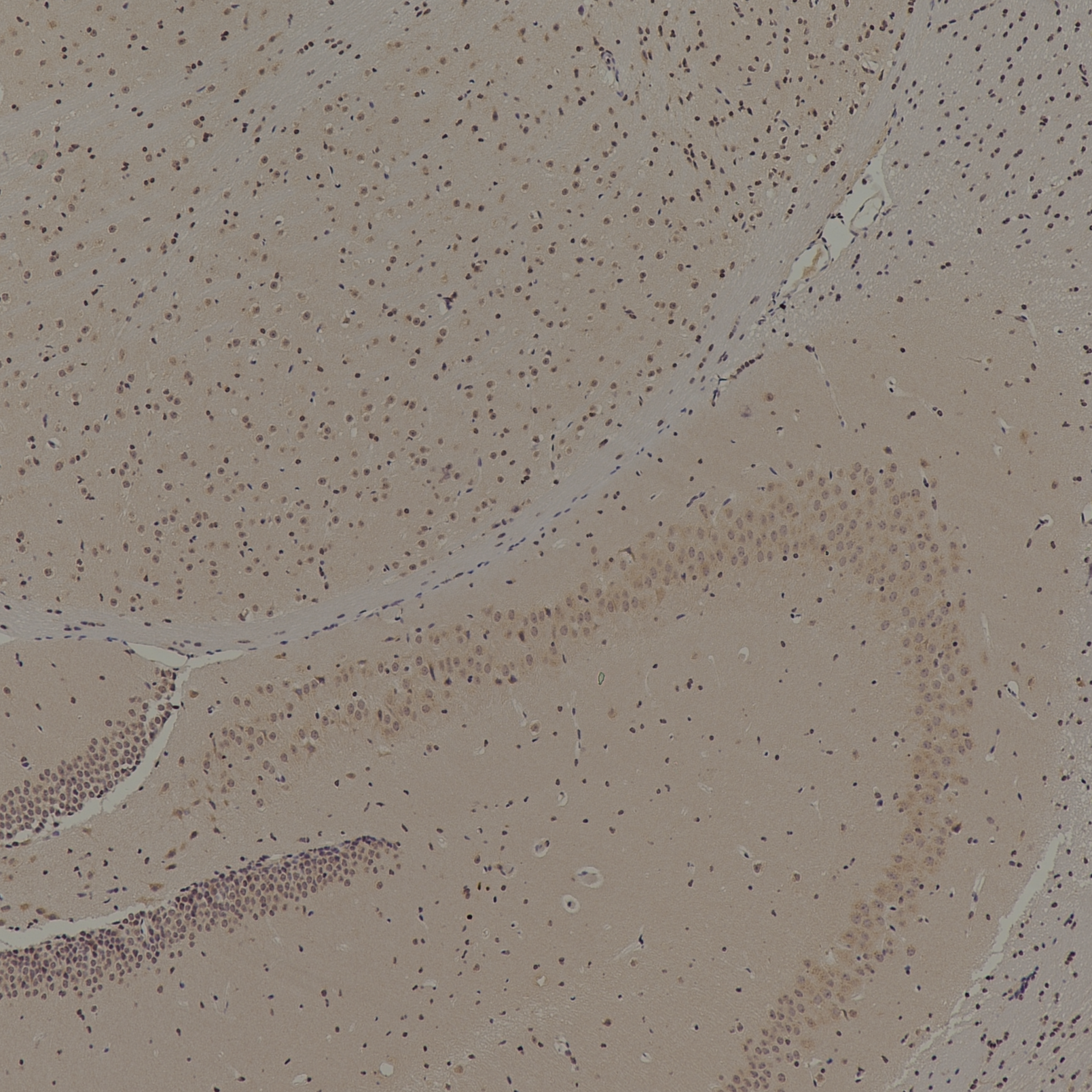

Supplement: S1 File — (ZIP) [file pone.0289248.s001.zip › data in brief/Immunohistochemistry/10x CRS+DFX for fig.2(a).png]

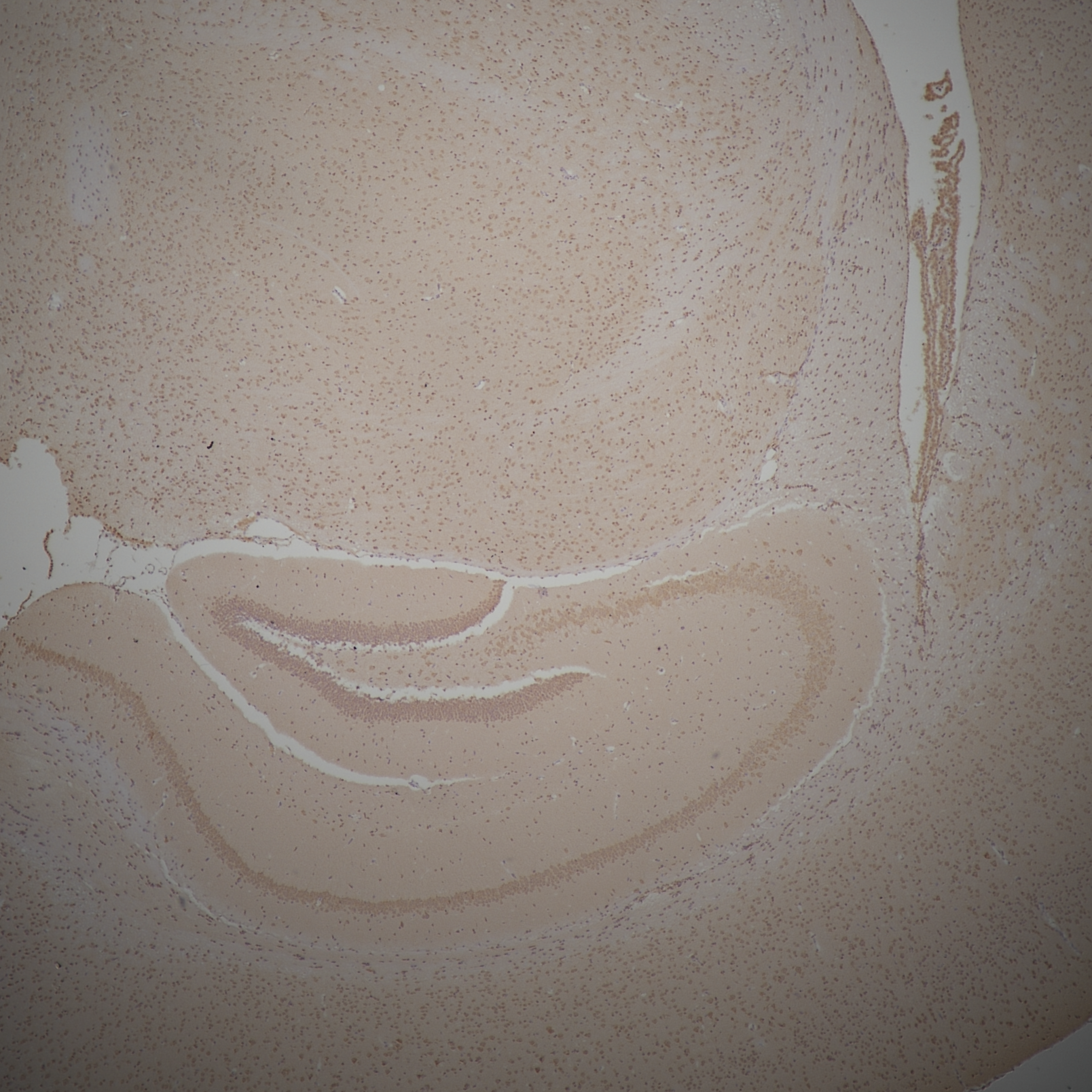

Supplement: S1 File — (ZIP) [file pone.0289248.s001.zip › data in brief/Immunohistochemistry/4x CON for fig.2(a).png]

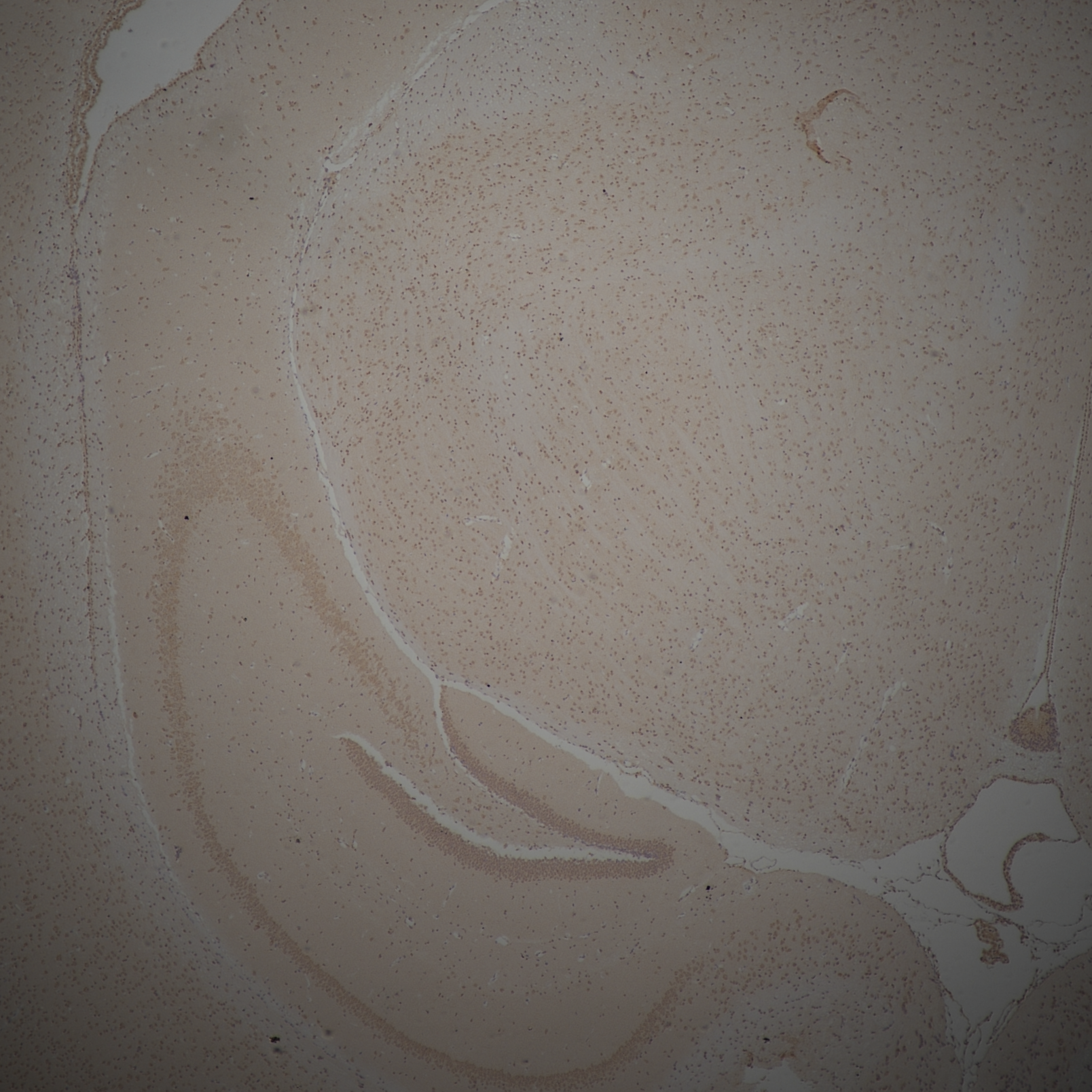

Supplement: S1 File — (ZIP) [file pone.0289248.s001.zip › data in brief/Immunohistochemistry/4x CRS for fig.2(a).png]

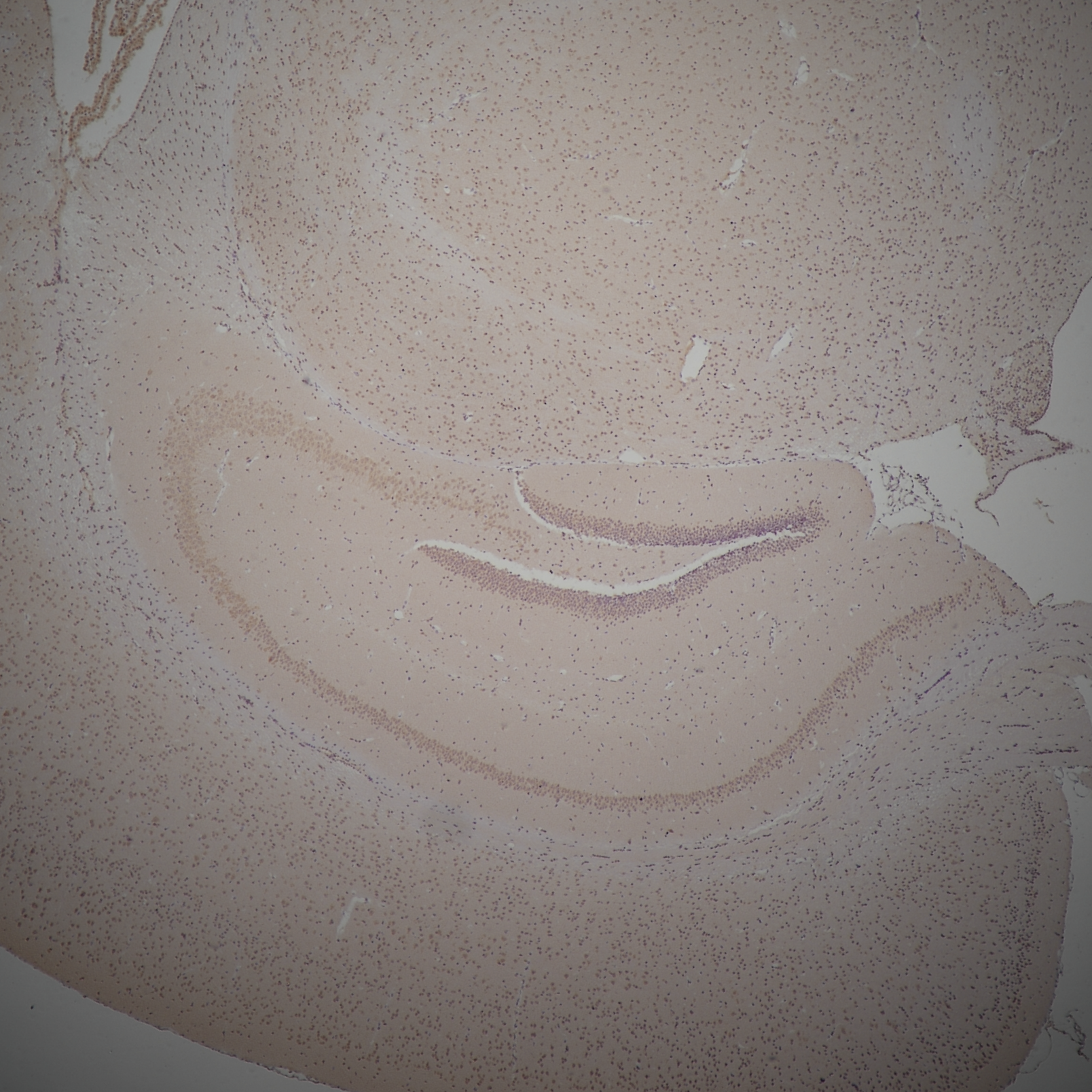

Supplement: S1 File — (ZIP) [file pone.0289248.s001.zip › data in brief/Immunohistochemistry/4x CRS+AGO for fig.2(a).png]

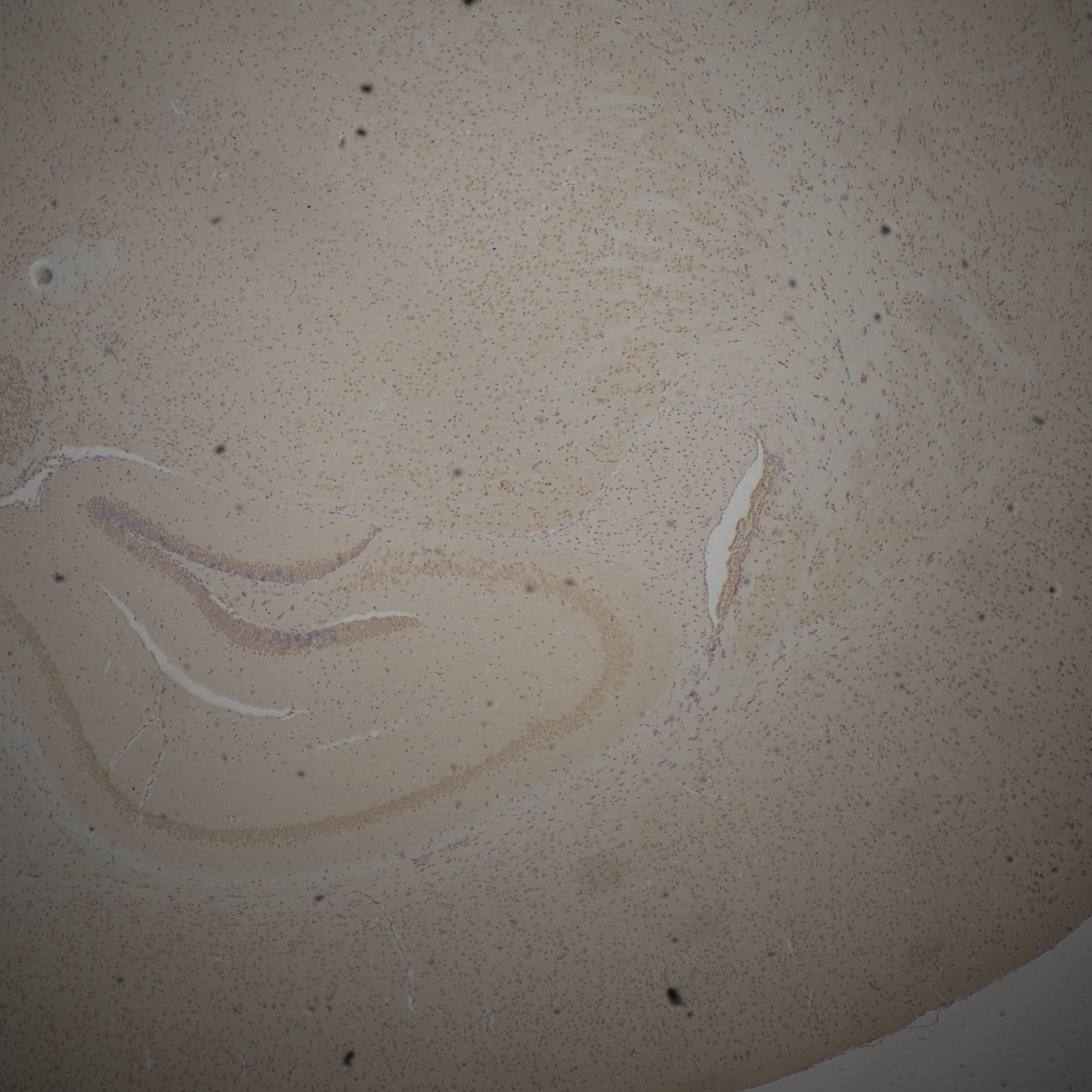

Supplement: S1 File — (ZIP) [file pone.0289248.s001.zip › data in brief/Immunohistochemistry/4x CRS+AGO+DFX for fig.2(a).png]

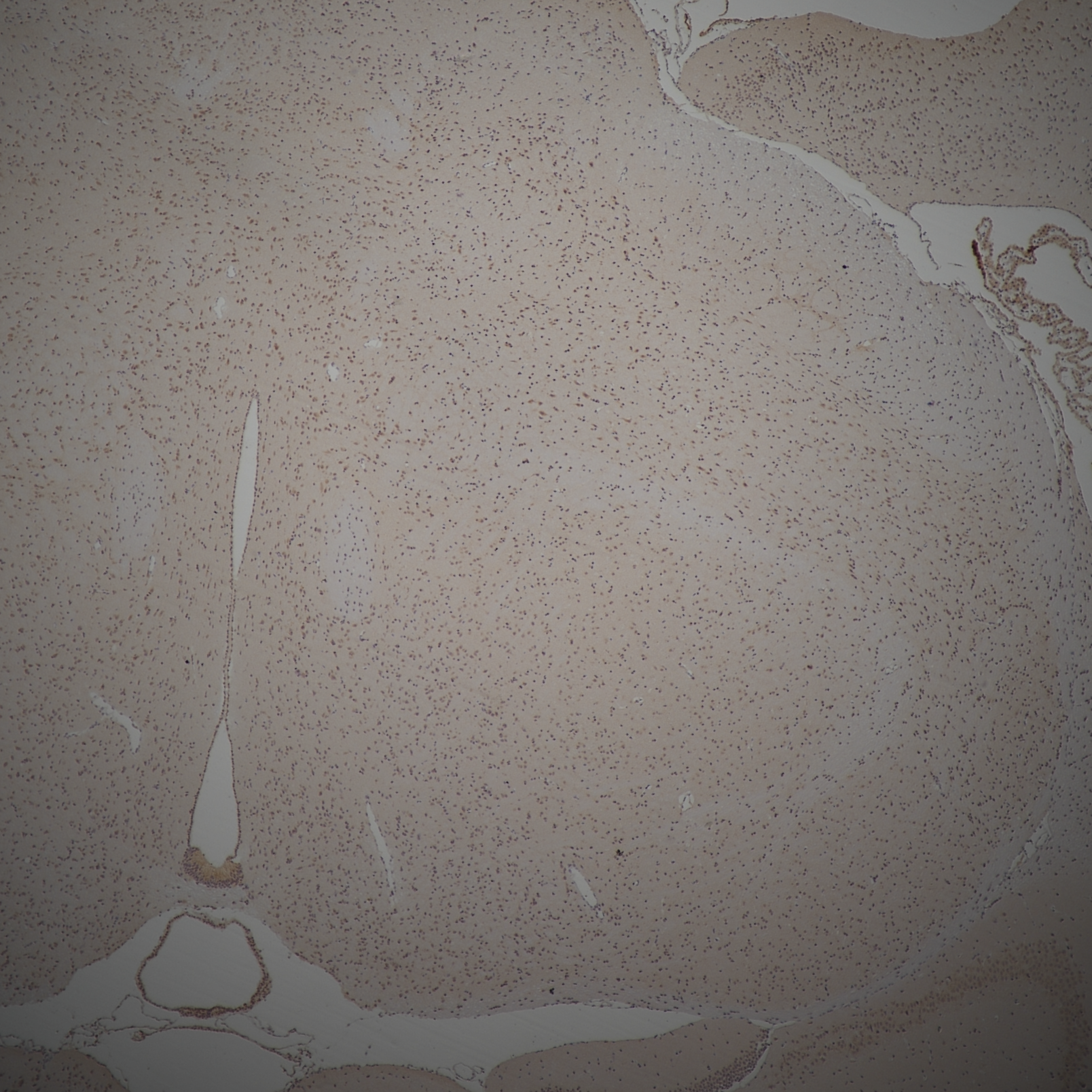

Supplement: S1 File — (ZIP) [file pone.0289248.s001.zip › data in brief/Immunohistochemistry/4x CRS+DFX for fig.2(a).png]

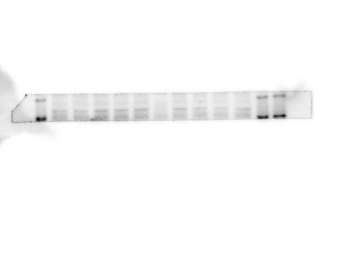

Supplement: S1 File — (ZIP) [file pone.0289248.s001.zip › data in brief/westernblot/Catalase(AGO) for Fig.3d.tif]

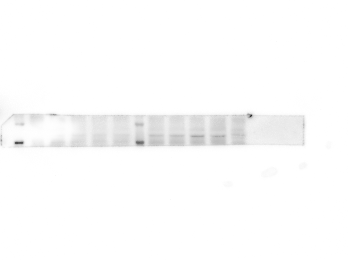

Supplement: S1 File — (ZIP) [file pone.0289248.s001.zip › data in brief/westernblot/Catalase(CON) for Fig.3d.tif]

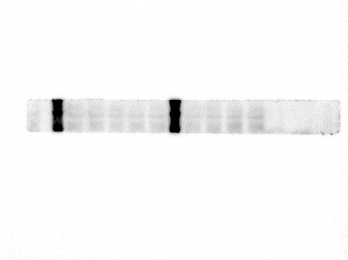

Supplement: S1 File — (ZIP) [file pone.0289248.s001.zip › data in brief/westernblot/Catalase(CRS) for Fig.3d.tif]

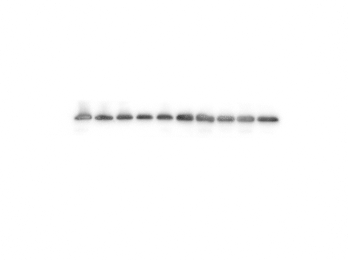

Supplement: S1 File — (ZIP) [file pone.0289248.s001.zip › data in brief/westernblot/GAPDH for Fig.3d.tif]

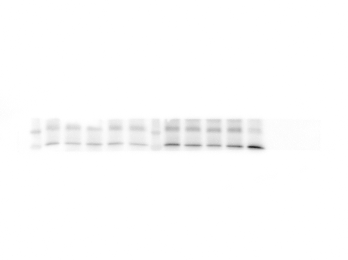

Supplement: S1 File — (ZIP) [file pone.0289248.s001.zip › data in brief/westernblot/IkBa(AGO) for Fig.3g.tif]

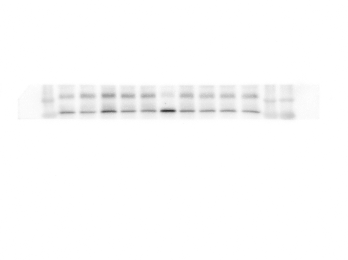

Supplement: S1 File — (ZIP) [file pone.0289248.s001.zip › data in brief/westernblot/IkBa(CON)&IkBa(CRS) for Fig.3g.tif]

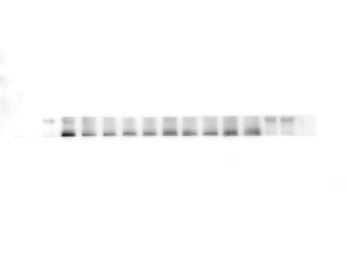

Supplement: S1 File — (ZIP) [file pone.0289248.s001.zip › data in brief/westernblot/NF-kB p65(CON) for Fig.3g.tif]

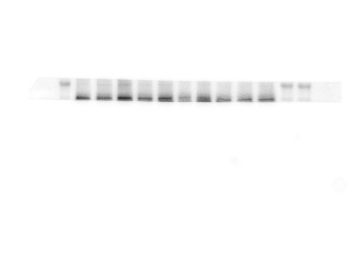

Supplement: S1 File — (ZIP) [file pone.0289248.s001.zip › data in brief/westernblot/NF-kB p65(CRS)&NF-kB p65(AGO) for Fig.3g.tif]
