# Supplementary material for: Agomelatine prevented depression in the chronic restraint stress model through enhanced catalase activity and halted oxidative stress
Source: PLoS One. 2024 Feb 9;19(2):e0289248. doi: 10.1371/journal.pone.0289248 (PMC10857580; doi:10.1371/journal.pone.0289248)
Supplement: Attachment — Submitted filename: Response to Reviewers.docx [file pone.0289248.s002.docx]

**Response Letter to Reviewers**

**Thanks very much for taking the time to review this manuscript and providing us with this great opportunity to submit a revised version of our manuscript (Manuscript: PONE-D-23-03416). We appreciate all your detailed comments and suggestions! We have incorporated them, and those changes are highlighted in blue within the re-submitted files. We hope this revised manuscript has addressed your concerns and look forward to hearing from you.**

**I have the following replies to the comments of reviewers.**

---------------------------------------------------------------------------------------------------------------------------

**# Comments from reviewer 1:**

The current work aimed to investigate the possible antidepressant effect of agomelatine (AGO) and the role of the catalase (CAT) enzyme in mediating this effect. In addition, the theories of increased oxidative stress and neuroinflammation were tested in chronic restraint stress model. The manuscript follows the standard scientific way; however, it needs much improvement. I have The following concerns about the manuscript:

**The shortcomings are as follows:**

**1) Title**

**It doesn’t express the results of the current study in a good way because 1) AGO was given from day 1 of the study and not after the development of the chronic restraint stress (CRS) model; 2) There were no significant differences regarding the inflammatory markers tested between all groups; 3) Blocking catalase by deferasirox didn’t worsen the CRS model. The authors could think of another title, I suggest “Agomelatine prevented depression in chronic restraint stress model through enhanced catalase activity and halted oxidative stress”. If significant results were found regarding the inflammatory markers after revising the post hoc analysis of ANOVA, you may express this in the title as well.**

**2) Abstract**

**• Page 2, Line 21 (first line): The abbreviation of agomelatine (AGO) should be mentioned and used subsequently after that, see also line 32 & 39.**

**3) Introduction**

**• Page 3, lines 52, 53: Please, modify the sentence “agomelatine (AGO) may exert its anxiolytic, depressive effects …” and choose a better word than “depressive” to express the drug-calming effect; otherwise, remove it. This is because “depressive” is not logical while the study aimed to test agomelatine in the treatment of depression.**

**• Page 3, line 56: Please, add references to the sentence stating “Previous studies”.**

**• Page 4, line 67: “glutathione” is not an antioxidant enzyme, please edit to “glutathione peroxidase”.**

**• Page 4, line 67: The word “neutralize” is better to be changed to “overcome”.**

**• Page 5, line 89: The Phrase “we propose a novel hypothesis” is not accurate since the relation between depression and increased ROS has been proposed many years ago. It is better to be edited to “Based on the proposed hypothesis of the involvement of oxidative stress in the pathogenesis of MDD, …”**

**• Page 5, line 90: Please, edit the phrase “AGO can improve depression” to “AGO may improve depression” so that the research question carries the possibility of the desired effect.**

**4) Results**

**• Page 6, line 117: Please, edit the “abovementioned trials” by adding a space after the word “above” and replacing “trials” with “tests”. Also, replace the word “trial” in line 118.**

**• Page 6, line 122: The phrase “through routes that enhance CAT activity” is not appropriate in place because, in this paragraph, you only express the behavioral test results and the results of oxidative stress have not been mentioned yet.**

**• Page 8, line 152: Please, delete “CAT inhibitor” because deferasirox is primarily classified as an iron chelator, and in the results, we only mention the effect of the tested drugs without explanation.**

**• Page 9, line 177: Please, edit this sentence “The results showed that the CRS group had significantly lower serum SOD and MDA concentrations after AGO treatment” as serum SOD was significantly increased with AGO treatment.**

**• Page 9, line 179: Please, remove this sentence “This is because CRS causes elevated levels of oxidative stress, and AGO was shown to attenuate oxidative stress in mouse models of depression, potentially because of its effects on CAT activity” as it is an explanatory sentence. It should be placed within the discussion.**

**• Page 9, line 185: NF-κB is a downstream regulator of inflammatory cytokines. Please, correct the word “upstream regulators”.**

**5) Discussion**

**• Page 10, line 203: This sentence is vague “This means that, except in melatonin and 5-HT2C, the synergistic effect of regulating oxidative stress may be another mode of action for AGO”. Please, edit the sentence to be more clear and express the desired aim.**

**• Page 11, line 228: The sentence “In turn, this leads to increased CAT expression…” should be edited and clarified whether you want to refer to the results of the CRS or AGO results. Also, revise the changes of CAT, SOD, and MDA accordingly.**

**• Page 12, line 246: The following inference is not correct “demonstrating that oxidative stress may be caused by the inhibition of CAT activity”. It refers that other mechanisms than CAT inhibition participating in the CRS model &/or MDD pathogenesis. Your inference could be correct if the administration of DFX further worse the CRS model. Please, explain or criticize.**

**• Page 13, line 259: Please, edit the sentence starting with “AGO blocked this activation …” to show that these are the result of a previous study (reference 39) and not the current study.**

**6) Conclusion**

**• Page 13, line 270: Please, edit the phrase “is the underlying antidepressant mechanism” to “is one of the underlying antidepressant mechanisms”.**

**7) Methods**

**• Page 13, line 272: “Study subjects” is better to be replaced with “Study animals”.**

**• Page 14 (Model construction and grouping): Please, add references to the paragraph on model establishing.**

**• Page 14, line 285: Please, check this phrase for grammar “Each mouse was fed water on an ad libitum diet”. I think the preposition should be removed “Each mouse was fed water ad libitum”.**

**• Page 14, line 286: Please, mention the criteria of “Successful mice” and a reference for that.**

**• Page 14, line 290: Please, correct the drug name “dilarosine” to “deferasirox”.**

**• Page 14, lines 291-297: Please, mention the references for the selected dose of the drugs. Also, mention the route of administration clearly and edit “saline infusion for gavage”. How did you calculate the dose of the saline (normal saline 10 mg/kg/day) while it is a solution?**

**• Page 15: Please, add references to the Sugar-water preference test, Open Field Test, and Forced swimming test.**

**• Page 16: Please, add references to Immunohistochemistry. Also, specify which part of the brain has been used in paraffin tissue sections.**

**• Page 17, line 340: Please, mention the catalog number and the scientific name of the ELISA kits used to assess CAT, SOD, and MDA.**

**• Page 17, line 342: Please, add the reference for CAT activity detection.**

**• Page 17, line 354: Please, mention the reference for the primers’ design.**

**• Page 18, line 381: It is illogical to use the T-test to compare between two groups' mean since SPSS contains post hoc tests after ANOVA. Please, recheck all your statistical analysis with the appropriate post hoc test again.**

**8) Figure legends**

**• Figure 1 legend (Page 6, line 124:) I suggest using the following title “Effects of the CRS, AGO, and DFX intervention on body weight and behavioral tests” and remove the phrase “Effects of the CRS, AGO, and DFX intervention” from the subsequent subtitle. Please, edit as appropriate and add the name of the statistical test used.**

**• Figure 2 legend (Page 7, line 143): Please, edit the title to “Effects of the CRS, AGO, and DFX intervention on immunohistochemistry of CAT protein in the hippocampus of mice” and add the name of the statistical test used.**

**• Figure 3 legend (Page 8, line 157): Please, add the name of the statistical test used.**

**9) English proof**

**• Please, the manuscript has to be checked by a native English speaker.**

**Thank you and best wishes.**

**# Authors response:**

Thank you very much for your time in reviewing the manuscript and your encouraging comments on the merits. We also appreciate your clear feedback and hope that you will find that the explanation has fully addressed all of your concerns and that this re-submitted manuscript is satisfactory. Below, we will specifically address the comments point-by-point, and changes are highlighted in blue within the re-submitted files.

**Comment 1: Title**

*”It doesn’t express the results of the current study in a good way because 1) AGO was given from day 1 of the study and not after the development of the chronic restraint stress (CRS) model; 2) There were no significant differences regarding the inflammatory markers tested between all groups; 3) Blocking catalase by deferasirox didn’t worsen the CRS model. The authors could think of another title, I suggest “Agomelatine prevented depression in chronic restraint stress model through enhanced catalase activity and halted oxidative stress”. If significant results were found regarding the inflammatory markers after revising the post hoc analysis of ANOVA, you may express this in the title as well.”*

**Response:**

Thank you for the detailed review to improve our manuscript. As suggested by the reviewer, We have revised the title to “**Agomelatine prevented depression in the chronic restraint stress model through enhanced catalase activity and halted oxidative stress**” to make it more accurate and scientific.

**Comment 2: Abstract**

*”• Page 2, Line 21 (first line): The abbreviation of agomelatine (AGO) should be mentioned and used subsequently after that, see also line 32 & 39.”*

**Response:**

Thank you for the detailed review to improve our manuscript. As suggested by the reviewer, we have added this important information to our re-submitted manuscript.

• Page 2, Line 21: **“Agomelatine (AGO) is an antidepressant with unique pharmacological effects”**

• Page 2, Line 32: **“Four weeks of AGO treatment significantly improved depression-like behavior in mice through the activation of catalase in the hippocampus and serum of the model mice”**

• Page 2, Line 39: **“AGO can exert antidepressant effects by altering oxidative stress by modulating scatalase activity. ”**

**Comment 3: Introduction**

*- Page 3, lines 52, 53: Please, modify the sentence “agomelatine (AGO) may exert its anxiolytic, depressive effects …” and choose a better word than “depressive” to express the drug-calming effect; otherwise, remove it. This is because “depressive” is not logical while the study aimed to test agomelatine in the treatment of depression.*

**Response:**

Thank you for pointing this out. We have revised it in our manuscript to reflect your great suggestion. (Page 3, lines 52, 53)

**“Agomelatine (AGO), an alternative antidepressant, may exert its anxiolytic effects through an agonist effect on MT1/MT2 receptors and an antagonist effect on 5-HT_2C_ receptors.”**

*- Page 3, line 56: Please, add references to the sentence stating “Previous studies”.*

**Response:**

Thank you for the detailed review to improve our manuscript. As suggested, we have added this important information to our re-submitted manuscript. (Page 3, line 56):

**“Previous studies have shown that the antidepressant effects of AGO are not entirely mediated through the same mechanisms as other conventional antidepressants [5-9].”**

**Reference:**

5. Chen F, Chen S, Liu J, Amin N, Jin W, Fang M. Agomelatine softens depressive-like behavior through the regulation of autophagy and apoptosis. BioMed Res Int. 2021;2021: 6664591. doi: [10.1155/2021/6664591](https://doi.org/10.1016/j.lfs.2018.09.003).

6. Lan T, Wu Y, Zhang Y, Li S, Zhu Z, Wang L, et al. Agomelatine rescues lipopolysaccharide-induced neural injury and depression-like behaviors via suppression of the Gαi-2-PKA-ASK1 signaling pathway. J Neuroinflammation. 2022;19: 117. doi: [10.1186/s12974-022-02479-x](https://doi.org/10.1016/j.lfs.2018.09.003).

7. Rebai R, Jasmin L, Boudah A. Agomelatine effects on fat-enriched diet induced neuroinflammation and depression-like behavior in rats. Biomed Pharmacother. 2021;135: 111246. doi: [10.1016/j.biopha.2021.111246.](https://doi.org/10.1016/j.lfs.2018.09.003)

8. Ozdamar Unal G, Demirdas A, Nazıroglu M, Ovey IS. Agomelatine attenuates calcium signaling and apoptosis via the inhibition of TRPV1 channel in the hippocampal neurons of rats with chronic mild stress depression model. Behav Brain Res. 2022;434: 114033. doi: [10.1016/j.bbr.2022.114033.](https://doi.org/10.1016/j.lfs.2018.09.003)

9. Rainer Q, Xia L, Guilloux JP, Gabriel C, Mocaër E, Hen R, et al. Beneficial behavioural and neurogenic effects of agomelatine in a model of depression/anxiety. Int J Neuropsychopharmacol. 2012;15: 321-335. doi: [10.1017/S1461145711000356.](https://doi.org/10.1016/j.lfs.2018.09.003)

*- Page 4, line 67: “glutathione” is not an antioxidant enzyme, please edit to “glutathione peroxidase”.*

*- Page 4, line 67: The word “neutralize” is better to be changed to “overcome”.*

**Response:**

We appreciate your valuable suggestion. We have revised our re-submitted manuscript to reflect your great suggestion. (Page 4, line 66)

**“Catalase (CAT), such as superoxide dismutase (SOD) and glutathione peroxidase, are antioxidative enzymes that can overcome the role of oxidative stress [14].”**

*- Page 5, line 89: The Phrase “we propose a novel hypothesis” is not accurate since the relation between depression and increased ROS has been proposed many years ago. It is better to be edited to “Based on the proposed hypothesis of the involvement of oxidative stress in the pathogenesis of MDD, …”*

*- Page 5, line 90: Please, edit the phrase “AGO can improve depression” to “AGO may improve depression” so that the research question carries the possibility of the desired effect.”*

**Response:**

Thank you for pointing this out. We have revised our re-submitted manuscript to reflect your great suggestion. (Page 5, lines 87–90)

**“The activation of the oxidative stress pathway is an important pathophysiological factor in depression, based on the proposed hypothesis of the involvement of oxidative stress in the pathogenesis of MDD. AGO may improve depression symptoms, anxiety symptoms, and biochemical indicators of depression by affecting oxidative stress and inflammation.”**

**Comment 4: Results**

*- Page 6, line 117: Please, edit the “abovementioned trials” by adding a space after the word “above” and replacing “trials” with “tests”. Also, replace the word “trial” in line 118.*

**Response:**

We appreciate your valuable suggestion. (Page 6, lines 114–117)

**“However, the DFX treatment and CRS groups did not show significant differences in the tests mentioned above. Unfortunately, no significant effects were observed in the total travel time and central region movement time in the OFT tests (Fig. 1g–1h). ”**

*- Page 6, line 122: The phrase “through routes that enhance CAT activity” is not appropriate in place because, in this paragraph, you only express the behavioral test results and the results of oxidative stress have not been mentioned yet.*

**Response:**

Thank you for the detailed review to improve our manuscript. As suggested, we have made these revisions to our re-submitted manuscript. (Page 6, line 119)

**“…, indicating that AGO partially improves depressive-like behavior in mice.”**

*- Page 8, line 152: Please, delete “CAT inhibitor” because deferasirox is primarily classified as an iron chelator, and in the results, we only mention the effect of the tested drugs without explanation.*

**Response:**

Thank you for pointing this out. We have revised our re-submitted manuscript to reflect your great suggestion. (Page 8, line 151)

**“CAT activity increased significantly after AGO treatment, and the DFX had a clear inhibitory effect.”**

*- Page 9, line 177: Please, edit this sentence “The results showed that the CRS group had significantly lower serum SOD and MDA concentrations after AGO treatment” as serum SOD was significantly increased with AGO treatment.*

*- Page 9, line 179: Please, remove this sentence “This is because CRS causes elevated levels of oxidative stress, and AGO was shown to attenuate oxidative stress in mouse models of depression, potentially because of its effects on CAT activity” as it is an explanatory sentence. It should be placed within the discussion.*

**Response:**

We appreciate your valuable suggestion. As suggested, we have revised it in our re-submitted manuscript.(Page 9, line 178 - 181)

**“The results showed that the CRS group had significantly lower serum MDA concentrations after AGO treatment, while serum SOD was increased significantly with AGO treatment. Meanwhile, this therapeutic effect was partially offset by DFX.”**

*- Page 9, line 185: NF-κB is a downstream regulator of inflammatory cytokines. Please, correct the word “upstream regulators”.”*

**Response:**

Thank you for the detailed review to improve our manuscript. We have revised the results according to your great suggestion, and the revised version can be found in the re-submitted files. (Page 9, line 185)

**“Extensive preclinical and clinical evidence shows that external stress is accompanied by increased proinflammatory cytokines and their downstream regulators.”**

**Comment 5: Discussion**

*- Page 10, line 203: This sentence is vague “This means that, except in melatonin and 5-HT2C, the synergistic effect of regulating oxidative stress may be another mode of action for AGO”. Please, edit the sentence to be more clear and express the desired aim.*

**Response:**

Thank you for pointing this out. We have revised our re-submitted manuscript to reflect your great suggestion. (Page 10, line 203)

**“…, meaning that regulating oxidative stress may be another mechanism of action for depression of AGO [27].”**

*- Page 11, line 228: The sentence “In turn, this leads to increased CAT expression…” should be edited and clarified whether you want to refer to the results of the CRS or AGO results. Also, revise the changes of CAT, SOD, and MDA accordingly.*

**Response:**

Thank you for pointing this out. We apologize for these mistakes. Accordingly, we have revised our re-submitted manuscript to reflect your great suggestion. (Page 11, line 227–228)

**“Meanwhile, CRS also leads to increased MDA expression in the serum, decreased CAT activity in the mouse hippocampus, and decreased SOD expression in the serum, …”**

*- Page 12, line 246: The following inference is not correct “demonstrating that oxidative stress may be caused by the inhibition of CAT activity”. It refers that other mechanisms than CAT inhibition participating in the CRS model &/or MDD pathogenesis. Your inference could be correct if the administration of DFX further worse the CRS model. Please, explain or criticize.*

**Response:**

Thank you for the detailed review to improve our manuscript. As suggested, we have revised our re-submitted manuscript. (Page 12, line 245)

**“It may be that CAT inhibition has participated in the pathological mechanism of CRS model.”**

*- Page 13, line 259: Please, edit the sentence starting with “AGO blocked this activation …” to show that these are the result of a previous study (reference 39) and not the current study.*

**Response:**

We appreciate your valuable suggestion. As per your suggestion, we have revised the discussion. The revised version can be found in the re-submitted files. (Page 13, line 257)

**“Research shows that AGO blocked this activation …”**

**Comment 6: Conclusion**

*- Page 13, line 270: Please, edit the phrase “is the underlying antidepressant mechanism” to “is one of the underlying antidepressant mechanisms”.*

**Response 6:**

Thank you for pointing this out. We apologize for these mistakes and have revised the phrase on Page 13, line 269: **“… is one of the underlying antidepressant mechanisms.”**

**Comment 7: Methods**

*- Page 13, line 272: “Study subjects” is better to be replaced with “Study animals”.*

**Response:**

We appreciate your valuable suggestion. (Page 13, line 272)

**“Study animals”**

*- Page 14 (Model construction and grouping): Please, add references to the paragraph on model establishing.*

**Response:**

Thank you for the detailed review to improve our manuscript. As suggested, we have revised our re-submitted manuscript.

Page 14 (Model construction and grouping): [45,46]

**References:**

45. Chiba S, Numakawa T, Ninomiya M, Richards MC, Wakabayashi C, Kunugi H. Chronic restraint stress causes anxiety- and depression-like behaviors, downregulates glucocorticoid receptor expression, and attenuates glutamate release induced by brain-derived neurotrophic factor in the prefrontal cortex. Prog Neuropsychopharmacol Biol Psychiatry. 2012;39: 112-119. doi: [10.1016/j.pnpbp.2012.05.018.](https://doi.org/10.1080/01480545.2019.1663209)

46. Jung J, Lee SM, Lee MJ, Ryu JS, Song JH, Lee JE, et al. Lipidomics reveals that acupuncture modulates the lipid metabolism and inflammatory interaction in a mouse model of depression. Brain Behav Immun. 2021;94: 424-436. doi: [10.1016/j.bbi.2021.02.003.](https://doi.org/10.1080/01480545.2019.1663209)

*- Page 14, line 285: Please, check this phrase for grammar “Each mouse was fed water on an ad libitum diet”. I think the preposition should be removed “Each mouse was fed water ad libitum”.*

**Response:**

We appreciate your valuable suggestion. (Page 14, line 284)

**“Each mouse was fed water ad libitum”**

*- Page 14, line 286: Please, mention the criteria of “Successful mice” and a reference for that.*

**Response:**

Thank you for pointing this out, and we apologize for not expressing the criteria of “Successful mice” clearly. We have considered the following aspects to ensure that the CRS model has been successfully built as much as possible:

(1) The weight of the mice after CRS modeling decreased significantly, with statistical significance.
(2) Through behavioral tests, it proved that mice after CRS modeling had depressive behavior. In this experiment, 1) the decreased sugar water preference rate in the sugar-water preference test, 2) the prolonged forced swimming rest time in the forced swimming test, 3) the decreased central total distance in the open field tests.[1,2]

**References:**

1. Yan HC, Cao X, Das M, et al. Behavioral animal models of depression. Neuroscience bulletin 2010;26: 327-337. doi: 10.1007/s12264-010-0323-7.

2. Campos AC, Fogaça MV, Aguiar DC, et al. Animal models of anxiety disorders and stress. Braz J Psychiatry. 2013;35: S101-111. doi: 10.1590/1516-4446-2013-1139.

*- Page 14, line 290: Please, correct the drug name “dilarosine” to “deferasirox”.*

**Response:**

Thank you for the detailed review to improve our manuscript. As suggested, we have revised it in our re-submitted manuscript. (Page 14, line 290)

**“CAT inhibitor (deferasirox)”**

*- Page 14, lines 291-297: Please, mention the references for the selected dose of the drugs. Also, mention the route of administration clearly and edit “saline infusion for gavage”. How did you calculate the dose of the saline (normal saline 10 mg/kg/day) while it is a solution?*

**Response:**

Thank you for pointing this out. And we are sorry for these problems. “Saline (normal saline 10 mg/kg/day)”was a typo. Mice from the CRS group were given equal physiological saline (about 0.2 mL/10 g) through intragastric administration in this study. We again apologize for the confusion caused. (Page 14, lines 291-297)

**“Animals were treated for 4 weeks through intragastric administration. CRS + AGO mice, CRS + DFX mice, and CRS + AGO + DFX mice were treated with AGO (Jiangsu Hausen Pharmaceutical Company) 50 mg/kg and/or DFX (Shanghai Bide Medical Technology Company) 10 mg/kg once a day, all drugs were dissolved in normal saline, administered as described in previous studies.^[1-2]^ From the first day until the fourth weekend. Mice from the CRS group were given equal normal saline. Mice in the CON and DFX groups were not treated with CRS stimulation. The CON group was administered equal amounts of normal saline once a day; meanwhile, the DFX group was administered DFX (10 mg/kg) once a day. ”**

**References:**

1. Chen F, Chen S, Liu J, et al. Agomelatine softens depressive-like behavior through the regulation of autophagy and apoptosis. BioMed Res Int. 2021;2021: 6664591. doi: 10.1155/2021/6664591.

2. Wu Y, Ran L, Yang Y, et al. Deferasirox alleviates DSS-induced ulcerative colitis in mice by inhibiting ferroptosis and improving intestinal microbiota. Life Sci. 2023;314: 121312. doi: 10.1016/j.lfs.2022.121312.

*- Page 15: Please, add references to the Sugar-water preference test, Open Field Test, and Forced swimming test.*

**Response:**

Thank you for the detailed review to improve our manuscript. As suggested, we have added this important information to our re-submitted manuscript. (Page 15)

**Sugar-water preference test[48], Open Field Test [49], Forced swimming test [50].**

**References:**

48. Tang J, Liang X, Dou X, et al. Exercise rather than fluoxetine promotes oligodendrocyte differentiation and myelination in the hippocampus in a male mouse model of depression. Translational psychiatry, 2021, 11(1): 622.DOI: 10.1038/s41398-021-01747-3.

49. Song AQ, Gao B, Fan JJ, et al. Nlrp1 inflammasome contributes to chronic stress-induced depressive-like behaviors in mice. J Neuroinflammation. 2020;17: 178. doi: 10.1186/s12974-020-01848-8.

50. Lin J, Song Z, Chen X, et al. Trans-cinnamaldehyde shows anti-depression effect in the forced swimming test and possible involvement of the endocannabinoid system. Biochem Biophys Res Commun. 2019;518: 351-356.D doi: 10.1016/j.bbrc.2019.08.061.

*- Page 16: Please, add references to Immunohistochemistry. Also, specify which part of the brain has been used in paraffin tissue sections.*

**Response:**

Thank you for pointing this out and we are sorry for not expressing this clearly. (Page 16)

**“Mice were perfused with 4% paraformaldehyde (PFA) after being perfused with 50 mL (±) of saline through the heart for immunohistochemistry study, and their brains were removed. The hippocampus was isolated and fixed in 4% paraformaldehyde for 3 days at 4 ℃ after that. Fixed Hippocampal tissue was embedded in paraffin and sectioned to 10μm thickness.”**

**References:**

[1] Cai M, Lee J H, Yang E J. Electroacupuncture attenuates cognition impairment via anti-neuroinflammation in an alzheimer's disease animal model[J]. Journal of neuroinflammation, 2019, 16(1): 264.DOI: 10.1186/s12974-019-1665-3.

[2] Chen F, Chen S, Liu J, et al. Agomelatine softens depressive-like behavior through the regulation of autophagy and apoptosis[J]. BioMed research international, 2021, 2021: 6664591.DOI: 10.1155/2021/6664591.

*- Page 17, line 340: Please, mention the catalog number and the scientific name of the ELISA kits used to assess CAT, SOD, and MDA.*

**Response:**

Thank you for the detailed review to improve our manuscript. As suggested, we have added this important information to our re-submitted manuscript. (Page 17, line 340)

**“Mouse catalase (CAT) ELISA Kit (ml037752, Enzyme-Linked Biotechnology), Mouse superoxide dismutase (SOD) ELISA kit (ml643059, Enzyme-Linked Biotechnology), and MDA(Malondialdehyde) ELISA Kit (E-EL-0060c, Elabscience) content were determined according to the manufacturer’s instructions.”**

*- Page 17, line 342: Please, add the reference for CAT activity detection.*

**Response:**

We appreciate your valuable suggestion. As suggested, we have added this important information to our re-submitted manuscript. (Page 17, line 342)

**“CAT activity detection”**

**References:**

1. Zhang X, Jing S, Lin H, et al. Anti-fatigue effect of anwulignan via the nrf2 and pgc-1α signaling pathway in mice]. Food Funct. 2019;10: 7755-7766. doi: 10.1039/c9fo01182j.

2. Kong D, Yan Y, He XY, et al. Effects of resveratrol on the mechanisms of antioxidants and estrogen in Alzheimer's disease. BioMed Res Int. 2019;2019: 8983752. doi: 10.1155/2019/8983752.

3. Wang ZX, Lian WW, He J, et al. Cornuside ameliorates cognitive impairments in scopolamine induced ad mice: Involvement of neurotransmitter and oxidative stress J Ethnopharmacol. 2022;293: 115252. doi: 10.1016/j.jep.2022.115252.

*- Page 17, line 354: Please, mention the reference for the primers’ design.*

**Response:**

Thank you for the detailed review to improve our manuscript. (Page 17, line 354)

| Gene | Forward primer（5’-3’） | Reverse primer（5’-3’） |
| --- | --- | --- |
| NF-κB^[1]^ | CCGCTCGAGCTATGGACGATCTGTTTCCCCTC | CGGAATTCACCTTAGGAGCTGATCTGAC |
| CAT^[2]^ | GCAGATACCTGTGAACTGTC | GTAGAATGTCCGCACCTGAG |
| IкBα^[3]^ | CACTCCATCCTGAAGGCTACCAA | AAGGGCAGTCCGGCCATTA |
| GAPDH^[4]^ | TGTGGGCATCAATGGATTTGG | ACACCATGTATTCCGGGTCAAT |

**References:**

1. Yuan YH, Sun JD, Wu MM, Hu JF, Peng SY, Chen NH. Rotenone could activate microglia through nfκb associated pathway. Neurochem Res. 2013;38: 1553-1560. doi: 10.1007/s11064-013-1055-7.

2. El Mouatassim S, Guérin P, Ménézo Y. Expression of genes encoding antioxidant enzymes in human and mouse oocytes during the final stages of maturation. Mol Hum Reprod. 1999;5: 720-725. doi: 10.1093/molehr/5.8.720.

3. Feng X, Wang H, Ye S,  Guan J, Tan W, Cheng S, et al. Up-regulation of microRNA-126 may contribute to pathogenesis of ulcerative colitis via regulating NF-kappaB inhibitor IκBα. PloS One. 2012;7: e52782. doi: 10.1371/journal.pone.0052782.

4. Zha H, Miao W, Rong W, Wang A, Jiang W, Liu R, et al. Remote ischaemic perconditioning reduces the infarct volume and improves the neurological function of acute ischaemic stroke partially through the miR-153-5p/TLR4/p65/IkBa signalling pathway. Folia Neuropathol. 2021;59: 335-349. doi: 10.5114/fn.2021.112127.

*- Page 18, line 381: It is illogical to use the T-test to compare between two groups' mean since SPSS contains post hoc tests after ANOVA. Please, recheck all your statistical analysis with the appropriate post hoc test again.”*

**Response:**

Thank you for the detailed review to improve our manuscript. We have revised the methods according to your great suggestion. (Page 18, line 387)

**“SPSS 25.0 software (IBM Corp., Armonk, NY, USA) was used for the statistical analysis. All data are presented as mean ± SEM. Multi-group overall comparisons were analyzed using one-way ANOVA and post hoc multiple comparisons (LSD method). A P-value of <0.05 was considered statistically significant.”**

**Comment 8: Figure legends**

*- Figure 1 legend (Page 6, line 124:) I suggest using the following title “Effects of the CRS, AGO, and DFX intervention on body weight and behavioral tests” and remove the phrase “Effects of the CRS, AGO, and DFX intervention” from the subsequent subtitle. Please, edit as appropriate and add the name of the statistical test used.*

**Response:**

Thank you for the detailed review to improve our manuscript. As suggested by the reviewer, we have revised the Figure legends in our re-submitted manuscript. (Page 6, line 124)

**“Fig. 1. Effects of the CRS, AGO, and DFX intervention on body weight and behavioral tests~~.~~ Depression-like behavior in mice induced by CRS. (a) Initial body weight of the mice~~.~~ (b) Body weight of the mice after different treatments. (c) Weight gain of the mice. (d) Sucrose preference rate in mice. (e) Forced swimming rest time in mice. (f) The total distance of mice in the central region of the open field test. (g) Total time of mice in the central region of the open field. (h) The total distance of mice in the open field test. *P<0.05; ** P<0.01; *** P<0.001; CRS, chronic restraint stress; AGO, agomelatine; DFX, deferasirox; ns, no significant difference. Data are expressed as the mean ± standard deviation and were analyzed by one-way ANOVA, followed by post hoc multiple comparisons (LSD method). n=10.”**

*- Figure 2 legend (Page 7, line 143): Please, edit the title to “Effects of the CRS, AGO, and DFX intervention on immunohistochemistry of CAT protein in the hippocampus of mice” and add the name of the statistical test used.*

**Response:**

Thank you for pointing this out. We have revised it in our re-submitted manuscript to your great suggestion. (Page 7, line 143):

“**Fig. 2. Effects of the CRS, AGO, and DFX intervention on immunohistochemistry of CAT protein in the hippocampus of mice.**”

**“Data are expressed as the mean ± standard deviation and were analyzed by one-way ANOVA, followed by post hoc multiple comparisons (LSD method). n=3.”**

*- Figure 3 legend (Page 8, line 157): Please, add the name of the statistical test used.”*

**Response:**

We appreciate your valuable suggestion. (Page 8, line 157)

**“Data are expressed as the mean ± standard deviation and were analyzed by one-way ANOVA, followed by post hoc multiple comparisons (LSD method). n=3-5.”**

**Comment 9: English proof**

*- Please, the manuscript has to be checked by a native English speaker.”*

**Response 9:**

Thanks for your great suggestion on improving the accessibility of our manuscript. We use the language-editing service provided by *Editage* to ensure a native English speaker has checked the manuscript.

Finally, we would like to take this opportunity to thank you again for all your time and for providing us with this excellent opportunity to improve the manuscript. We hope you will find this revised version satisfactory.

---------------------------------------------------------------------------------------------------------------------------

**# Comments from reviewer 2:**

The manuscript is discussing the molecular mechanisms underlying the anti-depressant effect of Agomelatine.

**some important questions need to be answered:**

**1-Where the blood samples taken are collected from?**

**2- What is the reference for the duration of the induction method?**

**3- How is the doses for the used drugs determined ?**

**# Authors response:**

Thanks very much for taking your time to review this manuscript and your positive comments. We really appreciate all your detailed comments and suggestions! According to your valuable comments, we have made corresponding changes and adjustments in the manuscript. Those changes are highlighted in blue within the re-submitted files. Please find my itemized responses in below and my revisions in the re-submitted files.

**Comment 1:**

*“1-Where the blood samples taken are collected from?”*

**Response 1:**

Thank you for pointing this out. We taken the blood through puncture of the retrobulbar venous plexus. The retroorbital blood-collection method is widely used in mice. This method consistently yields a reasonable blood volume of high quality.^[1-3]^

In this study, after behavior index detection, **the mice were anesthetized with 0.3% (0.15 ml/10 g) pentobarbital sodium by intraperitoneal injection. After anesthesia was deemed effective, blood was collected through puncture of the retrobulbar venous plexus, and approximately 0.2 ml of blood was collected in a labeled centrifuge tube and kept at 20 ℃ for 1–2 h. The blood was precooled to 4°C and centrifuged at 3000 rpm/min for 10 min**.(341-346)

**References:**

[1] Fernández I, Peña A, Del Teso N, et al. Clinical biochemistry parameters in c57bl/6j mice after blood collection from the submandibular vein and retroorbital plexus[J]. J Am Assoc Lab Anim Sci, 2010, 49(2): 202-206.

[2] Christensen S D, Mikkelsen L F, Fels J J, et al. Quality of plasma sampled by different methods for multiple blood sampling in mice[J]. Lab Anim, 2009, 43(1): 65-71.DOI: 10.1258/la.2008.007075.

[3] Francisco C C, Howarth G S, Whittaker A L. Effects on animal wellbeing and sample quality of 2 techniques for collecting blood from the facial vein of mice[J]. J Am Assoc Lab Anim Sci, 2015, 54(1): 76-80.

**Comment 2:**

*“2- What is the reference for the duration of the induction method?”*

**Response 2:**

Thank you for the detailed review to improve our manuscript. As suggested, we have added this important information in our re-submitted manuscript.

In this study, the mice were restrained in a transparent cylindrical tube (3 cm inner diameter, 10 cm long, with 8 ventilated holes approximately 0.6 cm in diameter) for **4 weeks** of CRS molding. This restricted the head and limb movement so that the head could only be moved slightly. The binding time was from **10:00 to 16:00 (6 h).**

Chronic restrain stress(CRS) is considered as a scientific and effective mouse depression model. The specific duration of modeling in this experiment was performed as previously reported.[1-2]

(figure is from reference[1])

**References:**

[1] Chiba S, Numakawa T, Ninomiya M, et al. Chronic restraint stress causes anxiety- and depression-like behaviors, downregulates glucocorticoid receptor expression, and attenuates glutamate release induced by brain-derived neurotrophic factor in the prefrontal cortex[J]. Prog Neuropsychopharmacol Biol Psychiatry, 2012, 39(1): 112-119.DOI: 10.1016/j.pnpbp.2012.05.018.

[2] Jung J, Lee S M, Lee M J, et al. Lipidomics reveals that acupuncture modulates the lipid metabolism and inflammatory interaction in a mouse model of depression[J]. Brain Behav Immun, 2021, 94: 424-436.DOI: 10.1016/j.bbi.2021.02.003.

**Comment 3:**

*“3- How is the doses for the used drugs determined ?”*

**Response 3:**

Thank you for the detailed review to improve our manuscript. In this study, animals were treated during 4 weeks through intragastric administration. AGO (Jiangsu Hausen Pharmaceutical Company) 50 mg/kg and/or DFX (Shanghai Bide Medical Technology Company) 10 mg/kg, all drugs were dissolved in normal saline. For agomelatine, doses were chosen based on its activity over this range of doses in previously tested models of depression such as the forced swim test, the sucrose preference test, open field test and the elevated plus maze. ^[1]^ For deferasirox, 10 mg/kg was the dose shown to be active in a previous paper .^[2]^

**References:**

[1] Chen F, Chen S, Liu J, et al. Agomelatine softens depressive-like behavior through the regulation of autophagy and apoptosis[J]. BioMed research international, 2021, 2021: 6664591.DOI: 10.1155/2021/6664591.

[2] Wu Y, Ran L, Yang Y, et al. Deferasirox alleviates dss-induced ulcerative colitis in mice by inhibiting ferroptosis and improving intestinal microbiota[J]. Life Sci, 2023, 314: 121312.DOI: 10.1016/j.lfs.2022.121312.

Last but not least, we would like to take this opportunity to thank you again for all your time involved and this great opportunity for us to improve the manuscript. We hope you will find this revised version satisfactory and look forward to hearing from you. We would be glad to respond to any further questions and comments that you may have.
