# Supplementary material for: Agomelatine prevented depression in the chronic restraint stress model through enhanced catalase activity and halted oxidative stress
Source: PLoS One. 2024 Feb 9;19(2):e0289248. doi: 10.1371/journal.pone.0289248 (PMC10857580; doi:10.1371/journal.pone.0289248)
Supplement: Attachment — Submitted filename: Response to Reviewers2.docx [file pone.0289248.s003.docx]

**Response Letter to Reviewers**

**Thanks very much for taking the time to review this manuscript and providing us with this great opportunity to submit a revised version of our manuscript (Manuscript: PONE-D-23-03416). We appreciate all your detailed comments and suggestions! We have incorporated them, and those changes are highlighted in yellow within the re-submitted files. We hope this revised manuscript has addressed your concerns and look forward to hearing from you.**

**I have the following replies to the comments of reviewers.**

---------------------------------------------------------------------------------------------------------------------------

**# Comments from reviewer 1:**

Thank you so much. The authors addressed most of my concerns, but minor changes should be considered:

- Page 11, line 230: Please, add the references of the “previous studies” mentioned.
- Page 14, line 286: Please, add the criteria of “Successful mice” with references to the revised manuscript.
- Page 17, line 354: Please, add the sequences of the primers’ and the references to the revised manuscript.

Best wishes.

**# Authors response:**

Thank you very much for your time in reviewing the manuscript and your encouraging comments on the merits. We also appreciate your clear feedback and hope that you will find that the explanation has fully addressed all of your concerns and that this re-submitted manuscript is satisfactory. Below, we will specifically address the comments point-by-point, and changes are highlighted in yellow within the re-submitted files.

**Comment 1:***” Page 11, line 230: Please, add the references of the “previous studies” mentioned.”*

**Response:**

Thank you for the detailed review to improve our manuscript. As suggested by the reviewer, we add the references of the “previous studies”. (Page 11, lines 231)

“Evidence accumulated over the extensive course of a previous study[40,41] supports the idea that the antidepressant effect of AGO is because of its synergistic melatoninergic and serotonergic effects [29].”

Reference:

40.	Millan MJ, Marin P, Kamal M, Jockers R, Chanrion B, Labasque M, et al. The melatonergic agonist and clinically active antidepressant, agomelatine, is a neutral antagonist at 5-HT(2C) receptors. Int J Neuropsychopharmacol. 2011;14(6):768-83. doi: 10.1017/s1461145710001045.

41.	Norman TR, Cranston I, Irons JA, Gabriel C, Dekeyne A, Millan MJ, et al. Agomelatine suppresses locomotor hyperactivity in olfactory bulbectomised rats: a comparison to melatonin and to the 5-HT(2c) antagonist, S32006. European journal of pharmacology. 2012;674(1):27-32. doi: 10.1016/j.ejphar.2011.10.010.

**Comment 2:** *”Page 14, line 286: Please, add the criteria of “Successful mice” with references to the revised manuscript.”*

**Response:**

Thank you for the detailed review to improve our manuscript. As suggested by the reviewer, we have added this important information to our re-submitted manuscript. (Page 14, lines 283-289)

“We have considered the following aspects to ensure that the CRS model has been successfully built as much as possible: The weight of the mice after CRS modeling decreased significantly, with statistical significance.Through behavioral tests, it proved that mice after CRS modeling had depressive behavior. In this experiment, 1) the decreased sugar water preference rate in the sugar-water preference test, 2) the prolonged forced swimming rest time in the forced swimming test, 3) the decreased central total distance in the open field tests.[49,50]”

References:

49.	Yan HC, Cao X, Das M, Zhu XH, Gao TM. Behavioral animal models of depression. Neurosci Bull. 2010;26(4):327-37. doi: 10.1007/s12264-010-0323-7.

50.	Campos AC, Fogaça MV, Aguiar DC, Guimarães FS. Animal models of anxiety disorders and stress. Revista brasileira de psiquiatria (Sao Paulo, Brazil : 1999). 2013;35 Suppl 2:S101-11. doi: 10.1590/1516-4446-2013-1139.

**Comment 3:***“Page 17, line 354: Please, add the sequences of the primers’ and the references to the revised manuscript.”*

**Response:**

Thank you for pointing this out. We have revised it in our manuscript to reflect your great suggestion. (Page 18, lines 366)

| Gene | Forward primer（5’-3’） | Reverse primer（5’-3’） |
| --- | --- | --- |
| NF-κB[59] | CCGCTCGAGCTATGGACGATCTGTTTCCCCTC | CGGAATTCACCTTAGGAGCTGATCTGAC |
| CAT[60] | GCAGATACCTGTGAACTGTC | GTAGAATGTCCGCACCTGAG |
| IкBα[61] | CACTCCATCCTGAAGGCTACCAA | AAGGGCAGTCCGGCCATTA |
| GAPDH[62] | TGTGGGCATCAATGGATTTGG | ACACCATGTATTCCGGGTCAAT |

References:

59.	Yuan YH, Sun JD, Wu MM, Hu JF, Peng SY, Chen NH. Rotenone could activate microglia through nfκb associated pathway. Neurochem Res. 2013;38: 1553-1560. doi: 10.1007/s11064-013-1055-7.

60.	El Mouatassim S, Guérin P, Ménézo Y. Expression of genes encoding antioxidant enzymes in human and mouse oocytes during the final stages of maturation. Mol Hum Reprod. 1999;5: 720-725. doi: 10.1093/molehr/5.8.720.

61.	Feng X, Wang H, Ye S,  Guan J, Tan W, Cheng S, et al. Up-regulation of microRNA-126 may contribute to pathogenesis of ulcerative colitis via regulating NF-kappaB inhibitor IκBα. PloS One. 2012;7: e52782. doi: 10.1371/journal.pone.0052782.

62.	Zha H, Miao W, Rong W, Wang A, Jiang W, Liu R, et al. Remote ischaemic perconditioning reduces the infarct volume and improves the neurological function of acute ischaemic stroke partially through the miR-153-5p/TLR4/p65/IkBa signalling pathway. Folia Neuropathol. 2021;59: 335-349. doi: 10.5114/fn.2021.112127.

Finally, we would like to take this opportunity to thank you again for all your time and for providing us with this excellent opportunity to improve the manuscript. We hope you will find this revised version satisfactory.
